# The Lyme Borreliosis Spatial Footprint in the 21st Century: A Key Study of Slovenia

**DOI:** 10.3390/ijerph182212061

**Published:** 2021-11-17

**Authors:** Daša Donša, Veno Jaša Grujić, Nataša Pipenbaher, Danijel Ivajnšič

**Affiliations:** 1 Faculty of Natural Science and Mathematics, University of Maribor, Koroška cesta 160, 2000 Maribor, Slovenia; veno.grujic@um.si (V.J.G.); natasa.pipenbaher@um.si (N.P.); dani.ivajnsic@um.si (D.I.); 2 Faculty of Education, University of Maribor, Koroška cesta 160, 2000 Maribor, Slovenia; 3 Faculty of Arts, University of Maribor, Koroška cesta 160, 2000 Maribor, Slovenia

**Keywords:** CART, climate change, MGWR, Lyme disease, infection risk, spatial modelling

## Abstract

After mosquitoes, ticks are the most important vectors of infectious diseases. They play an important role in public health. In recent decades, we discovered new tick-borne diseases; additionally, those that are already known are spreading to new areas because of climate change. Slovenia is an endemic region for Lyme borreliosis and one of the countries with the highest incidence of this disease on a global scale. Thus, the spatial pattern of Slovenian Lyme borreliosis prevalence was modelled with 246 indicators and transformed into 24 uncorrelated predictor variables that were applied in geographically weighted regression and regression tree algorithms. The projected potential shifts in Lyme borreliosis foci by 2050 and 2070 were calculated according to the RCP8.5 climate scenario. These results were further applied to developing a Slovenian Lyme borreliosis infection risk map, which could be used as a preventive decision support system.

## 1. Introduction

Tick-borne diseases are increasingly important in the field of human public health in Europe [1]. A wide range of viruses, bacteria, and parasites that can potentially cause serious health problems in humans and other animals are transmitted by ticks [2]. Ticks are hematophagous ectoparasites and are, after mosquitoes, the most important vectors of various infectious diseases [3]. The tick becomes infected with the pathogen while feeding on the infected host and then transmits the pathogens to its next host. Thus, ticks harm humans obliquely, through the transmission of pathogenic organisms during feeding [4].

Ticks belong to the phylum Arthopoda, class Arachnida, subclass Acari (mites and ticks), and order Ixodida. The order Ixodida consists of three families: hard ticks (Ixodidae), soft ticks (Argasidae) and the monotypic family, Nuttalliellidae. Because of their characteristics (e.g., specialized host-seeking behavior, strong affinity for humans or a high responsiveness to stimuli that indicate the presence of hosts) and high abundance, the members of the hard tick family are good vectors of infectious diseases [5]. In Europe, the predominant species of the hard tick family is the castor bean tick (*Ixodes ricinus*), also known as the sheep tick. Compared to other species, the castor bean tick is very sensitive to ambient temperature and humidity. Furthermore, all developmental stages of this species are prone to desiccation, making air temperature and humidity even more important environmental limiting factors. For survival, at least 70–80% relative humidity is required, so castor bean ticks often return to lower-lying and more humid areas during their host search, allowing them to rehydrate [1]. Therefore, the presence of this species is usually limited to leaf fall and lower-lying vegetation in deciduous or mixed forests. However, in areas with more precipitation, they can also be found in coniferous forests and open habitats such as meadows and pastures [4]. Other important habitats include shrubs, forest edges, forest clearings, parks and gardens [6]. The castor bean tick is a major carrier of pathogens dangerous to both humans and other animals [7]. Among other diseases, this tick transmits the agent of tick-borne encephalitis and Lyme borreliosis (LB). The latter is considered to be the most common tick-borne disease in the Northern hemisphere [8]. In Europe, it is usually present in areas with a higher proportion of forests. The highest rate of reported cases occurs in Austria, Germany, Slovenia and Sweden [9]. In many European countries, including Slovenia, the incidence of LB is increasing every year [10].

One of the most frequently discussed, but perhaps less understood, consequences of global climate change is its effect on infectious diseases and human health [11]. Since ticks are ectothermic animals, changes in temperature, precipitation, humidity and other environmental conditions affect their development, behavior and population dynamics, as well as the development of the pathogens within them. Therefore, climate change indirectly affects the dynamics of diseases transmitted by ticks, which can further endanger human health [12]. The dispersion of ticks to higher latitudes and altitudes is correlated with global warming, and has already been recorded in Sweden [13], Norway [14], the Czech Republic [15] and the UK [16]. With climate and other environmental change processes, the tick-borne disease infection risk is expected to escalate. Bouchard et al. [17] concluded that ticks and tick-borne pathogens will become more abundant and active in the coming years.

This research addresses the territory of the Republic of Slovenia, where LB is the most common tick-borne disease. A large part of the country has been an endemic area for this disease for more than 70 years, and it should be emphasized that the incidence of LB is constantly increasing. Moreover, Slovenia is among the top European countries regarding the number of people infected with LB [18]. In the last decade, between 5000 and 7000 cases of LB infection were recorded in Slovenia annually [19]. The most common clinical forms among documented LB cases in Slovenia were erythema migrans (6000 cases reported, on average, between 2014 and 2018), meningitis (11 cases reported, on average, between 2014 and 2018) and polyneuropathy (4 cases reported, on average, between 2014 and 2018) [20]. In order to bridge the existing gaps in our understanding of LB distribution, we addressed the following research questions: (1) Are there significant spatial foci of LB in Slovenia? (2) Can these foci be explained with geospatial modelling techniques and proxy predictor variables describing natural and anthropogenic processes in the study area? Finally, (3) will climate change indirectly affect the spatial distribution of this disease in Slovenia?

## 2. Materials and Methods

### 2.1. The Dependent Variable

In order to reveal the spatial pattern of LB in Slovenia, we considered the average number of people infected with LB between 2015 and 2018 in each Slovenian municipality as the dependent variable. These data were obtained from the National Institute of Public Health [21]. However, since Slovenian municipalities differ in both spatial dimension and population density, we undertook, in the initial step, a normalization procedure for the dependent variable. Here, the average value of people infected with LB within each municipality between 2015 and 2018 was divided by the average number of inhabitants per municipality. The resulting normalized dependent variable, which represented LB prevalence (Y_LB_) in the study area, was then used in all further analyses.

### 2.2. Independent Variables

As independent variables, we used 238 separate indicators that could potentially explain the spatial distribution of infected castor bean ticks and, indirectly, the distribution of LB.

The Slovenian municipalities’ vector database and the digital elevation model (DEM) (25 m horizontal resolution) were obtained from the online platform e-Geodetic data belonging to the Surveying and Mapping Authority of the Republic of Slovenia [22].

To estimate vegetation density, the MODIS-based Normalized Difference Vegetation Index (NDVI) was obtained from the EarthData database [23] for the 2015–2018 period. In the following step, its average value, standard deviation and the linear trend coefficient were calculated in the ArcGIS environment [24,25].

To assess the indirect impact of land use on the prevalence of LB, we used 12 independent variables regarding land use types, which describe the potential habitat of the castor bean tick (Table S1). Land use vector data for 2017 were obtained from the Slovenian database MKGP-portal [26] and from the European database Land Copernicus [27] for 2018. The same database was used to download the Small Woody Features variable for the 2018 time window. For modelling purposes, all categorical land use variables were transformed to distance matrices by applying the Euclidean distance algorithm in the ArcGIS environment [24].

Another important proxy indicator potentially explaining the spatial distribution of tick-borne Lyme disease in Slovenia was the tick hosts. Here, the roadkill data from the Slovenia Forest Service [28] for the 2010–2018 time window were obtained. We classified these data into 5 categories, which included all roadkill species that could potentially represent castor bean tick hosts: birds, rodents, carnivores, ungulates and rabbits. For further analysis, we used the total number of roadkill in each category, considering the area of the individual municipality.

Since direct human-to-tick contact is needed to become infected, we also included some socio-economic drivers. These were obtained from the STAGE database [29], from the state administration database GOV.SI [30], and from the SiStat database [31]. We calculated the average number of inhabitants and the average population density per municipality in the time span 2015–2018. In addition, the average number of inhabitants who achieved higher or post-secondary education was also included. Next, the geometric mean of the population-aging index per municipality for the time range 2015–2018 and the harmonic mean value of the municipal development coefficient for the period 2015–2019 were added to the predictor list. To assess the impact of population movements on the risk of becoming infected with tick-borne Lyme disease, we used the average number of domestic tourist overnight stays in each municipality for the period 2015–2018, the geometric mean of the labor migration index for the period 2015–2018, and current road network vector data [22].

Climate predictors were obtained from the CHELSA website [32]. Current (1970–2013) and future (2050 and 2070) bioclimatic characteristics were considered. To encapsulate the variability of future bioclimatic predictions, data were analyzed from 5 significantly different global climate models (HadGEM2-ES (Hadley Center Global Environment Model version 2—Earth System configuration), CCSM4 (The Community Climate System Model 4), MIROC-ESM (MIROC Earth System Model), HadGEM2-CC (Hadley Center Global Environment Model version 2—low top configuration) and MPI-ESM-LR (The Coupled Max Planck Institute Earth System Model at base resolution)) [33], within the RCP 8.5 climate scenario.

### 2.3. Hot Spot Analysis

In order to inspect whether high or low Y_LB_ values were clustered by location, a spatial Cluster and Outlier Analysis (Anselin Local Morans I) was performed in ArcGIS [24]. However, a Hot Spot Analysis (Gets-Ord Gi*) was needed to check whether the aggregation of normalized values was statistically significant. Here, the contiguity–edges–corners conceptualization of spatial relationships was selected since we operated with polygon features and a normalized dependent variable (Y_LB_). In doing so, we were primarily interested in spatial clusters of high Y_LB_ values, i.e., those foci that indicated a much higher risk of infection with LB.

### 2.4. Preprocessing of Independent Variables

To avoid predictor multicollinearity, a correlation analysis in the R statistical software [34] was initially performed. Since not all our input independent variables met the normal distribution criteria, the Spearman correlation coefficient was applied. Thus, all redundant (positively [r_S_ > 0.6] and negatively correlated [r_S_ < −0.6]) predictors, except elevation, NDVI, climatic principal components and some land use types, were omitted in the modelling procedure (Table S1). These exceptions were necessary for Y_LB_ future modeling; nevertheless, their variance inflation factor (VIF) value was below the critical value of 5.

To reduce the number of bioclimatic predictors and simultaneously retain their informative value, we performed a principal component analysis in the ArcGIS environment [24]. The resulting first two principal components carried 80% of bioclimatic variability. This methodological procedure was then repeated for each of the 5 considered global climate models, for the time windows 2050 and 2070 within the RCP8.5 scenario. Finally, the zonal statistics algorithm in the ArcGIS software [24] was used to calculate the mean value of the first and second bioclimatic principal components per municipality for all time windows (current, 2050 and 2070).

Highly correlated roadkill categories (frequency of birds, rodents, carnivores, ungulates and rabbits) were transformed using Factor Analysis in the R statistical software [34]. Two factors explaining 50% of variance were then used in further analysis. The same was procedure was conducted with the following socio-economic predictors: average number of inhabitants, average population density, mean value of domestic tourist overnight stays, average number of inhabitants with higher and post-secondary education, and mean labor-migration index. Here, both further considered factors loaded 63% of the variability.

Thus, the final predictor list included 24 variables (Table S1).

### 2.5. Modelling

We calibrated, compared and, finally, averaged two models that enabled count data prediction: the Multivariate Geographically Weighted Regression (MGWR) and the machine learning-based Regression Tree Analysis (CART). The first one was developed with the MGWR 2.2 software [35] (Model Type = Poisson; Spatial Kernel = Adaptive; Bandwidth Searching = Golden Section) (Tables S2 and S3), and the second one with the rpart [36], rpart.plot [37], caTools [38] and tree [39] packages within the R statistical environment [34] (Figure S1). In order to properly specify regression trees, we divided our sample (212 municipalities) into test data (25%) and training data (75%). The resulting CART model was additionally pruned by applying the complexity parameter value with the smallest relative error (cp = 0.052). Based on the functional relationships between the dependent (Y_LB_) and predictor variables (Table S1) in the test data sample, we predicted the normalized number of infected people in the training data sample for current and future bioclimatic conditions (2050 and 2070). Essentially, both algorithms were used to predict future Y_LB_, and thus predict potential shifts in LB foci in Slovenia. In order to test and compare model quality, the resulting standardized residuals were tested for significant spatial autocorrelation with Moran’s I index. However, additional model quality indicators were also calculated: the Monte Carlo Spatial Variability test for the MGWR model, Explained Deviance (ED), Mean Absolute Error (MAE), and Root Mean Square Error (RMSE), Corrected Akaike Information Criterion (AICc).

### 2.6. Downscaling

After properly specifying both models, the resulting algorithms were used to generate an LB infection risk map of Slovenia, concerning climate change scenario RCP8.5 and time windows 2050 and 2070, in a higher spatial resolution. Local regression predictor coefficients and the Hot Spot analysis confidence level bin field (Gi_Bin) enabled the downscaling procedure within the MGWR model. Variable importance information and the aforementioned Gi_Bin value were used to downscale our dependent variable Y_LB_ with the CART model. Thus, we were able to spatially improve our results to the resolution of the weakest dynamic predictor (both bioclimatic principle components = 30 arcseconds).

## 3. Results

### 3.1. Foci of LB in Slovenia

**Figure 1:**
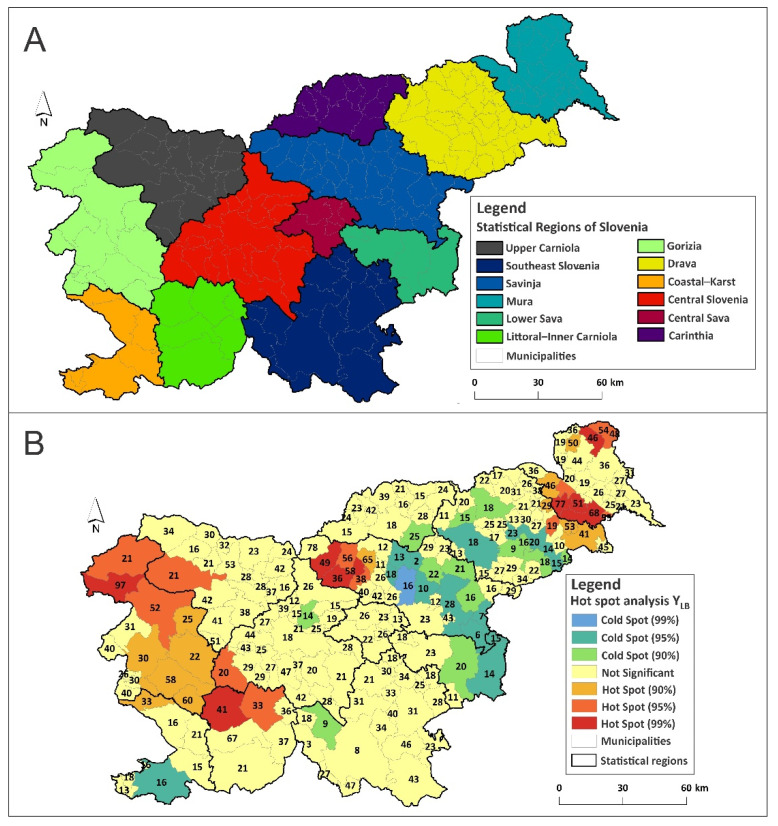
(**A**) Statistical regions of Slovenia (NUTS 3); (**B**) hot and cold spots for Y_LB_ in Slovenia. The number represent actual Y_LB_ values.

The Hot Spot Analysis of Y_LB_ revealed five statistically significant clusters of high values in Slovenia. One focus was in the northern part of the Mura Statistical Region; the second focus covered the eastern part of the Drava Statistical Region, and the third focus was located in the southwestern part of the Savinja Statistical Region. The fourth focus was located in the western part of the Upper Carniola Statistical Region and in northern parts of the Gorizia Statistical Region. The fifth focus was identified in the southern part of the Gorizia Statistical Region, in the northern part of the Coastal-Karst and Littoral–Inner Carniola Statistical Region, and in the southwestern part of the Central Slovenia Statistical Region (Figure 1A,B).

### 3.2. MGWR Modelling

**Figure 2:**
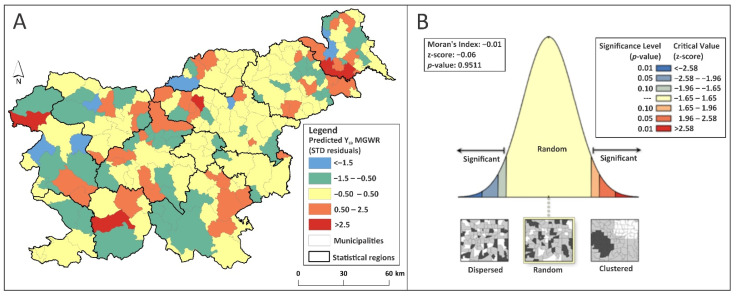
(**A**) Standardized residuals of predicted Y_LB_ (MGWR model); (**B**) The corresponding spatial autocorrelation test.

Before drawing any conclusions or making further predictions, the model’s over- and under-predictions were analyzed (Figure 2A,B). The insignificant (*p* > α; α = 0.05) Moran’s Index indicted that the MGWR model was properly specified, since its standardized residuals were normally distributed and free of spatial autocorrelation. The explained deviance reached 65% and the AICc value decreased from 1180.45 in the global regression, to 758.61 in the MGWR procedure (Tables S2 and S3).

**Figure 3:**
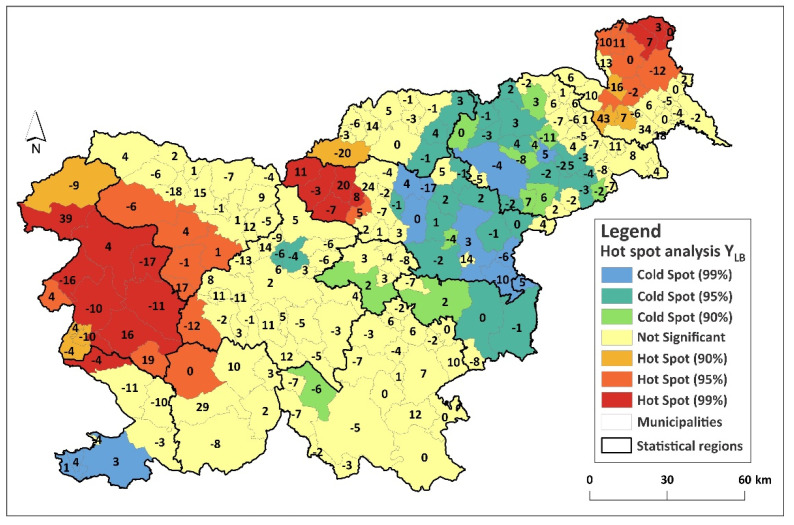
MGWR model predicted LB hot and cold spots. The numbers represent the deviations (residuals) of the predicted Y_LB_ from the actual Y_LB_.

The Monte Carlo Spatial Variability test indicated that the following predictors had statistically significantly varying estimates: elevation, land use (distance to Small Woody Features) and climatic conditions represented by the first and second principle components. These predictor variables had, in some places, a negative and, in other places, a positive effect on the dependent variable Y_LB_. The Hot Spot Analysis of predicted Y_LB_ enabled additional quality control, since we were thus able to compare real and predicted LB foci in Slovenia. The MGWR model predicted three major high value clusters of Y_LB_ (Figure 3). The value between the Mura and Drava Statistical Regions was slightly underestimated, and the value extending across three statistical regions (the southern part of the Gorizia Statistical Region, the northern part of the Coastal-Karst and Littoral–Inner Carniola Statistical Region, and the southwestern part of the Central Slovenia Statistical Region) was somewhat overestimated. However, all deviations from real Y_LB_ values are shown with numbers in Figure 3. Moreover, significantly low Y_LB_ value clusters were predicted with greater accuracy. However, other model performance parameters also confirmed its high quality (ED = 52%; MAE = 4.1 RMSE = 3.8; AICc improvement = 426.8).

### 3.3. CART Modelling

**Figure 4:**
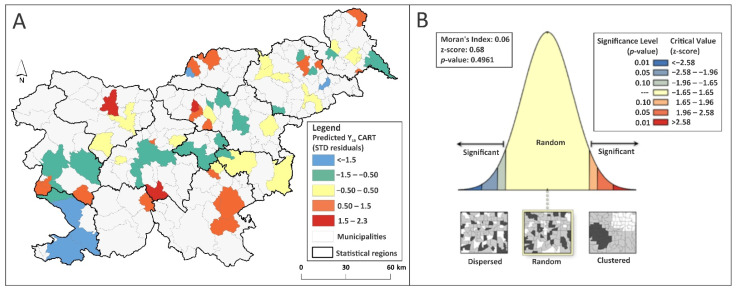
(**A**) CART predicted standardized residuals of Y_LB_ test data; (**B**) The corresponding spatial autocorrelation test.

The selected predictor variables implemented in the pruned CART model also yielded results with randomly dispersed over- and under-predictions, free of spatial autocorrelations (insignificant Moran’s I; *p* > α, α = 0.05; ED = 62%; MAE = 3.6 RMSE = 3.3) (Figure 4A,B). Here, both bioclimatic principal components were important decision makers for explaining the spatial pattern of Y_LB_ in Slovenia. However, the following predictors also significantly boosted model performance: elevation (14%), land use (dry open land (14%), pasture (9%), and extensive orchards (6%)), and NDVI (10%) (Figure S1).

**Figure 5:**
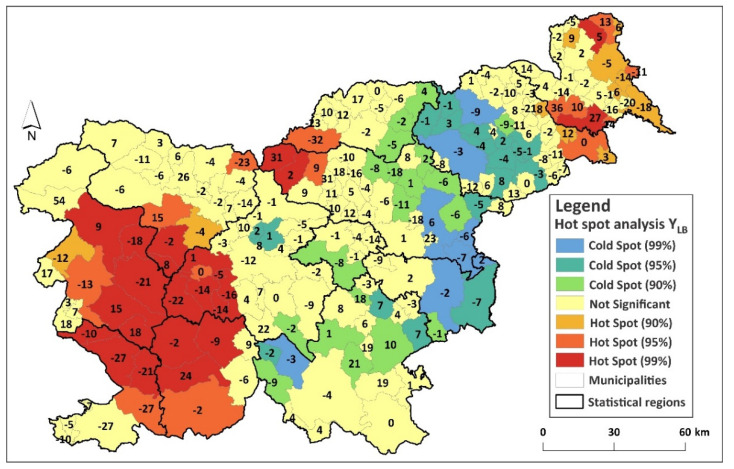
CART-predicted LB hot and cold spots. The numbers represent the deviations (residuals) of the predicted Y_LB_ from the actual Y_LB_.

Four LB foci were identified by calculating CART-predicted Y_LB_ hot and cold spots. Thus, better results were produced in the northeastern part of the Mura Statistical Region and in the eastern part of the Drava Statistical Region (Figure 5). The aggregation of predicted high values was partly in accordance with actual Y_LB_ hot spots in the northern part of the Littoral–Inner Carniola, in the western part of Central Slovenia and in the eastern part of the Gorizia Statistical Region. However, both Y_LB_ foci in the eastern part of Slovenia were slightly overpredicted. The opposite situation can be observed in the northern and western parts of the study area, where most municipalities identified as LB hot spots had underpredicted Y_LB_ values. Additional similarities with the MGWR model results were identified in the predicted quality of Y_LB_ cold spot distribution.

### 3.4. LB Infection Risk Assessment

**Figure 6:**
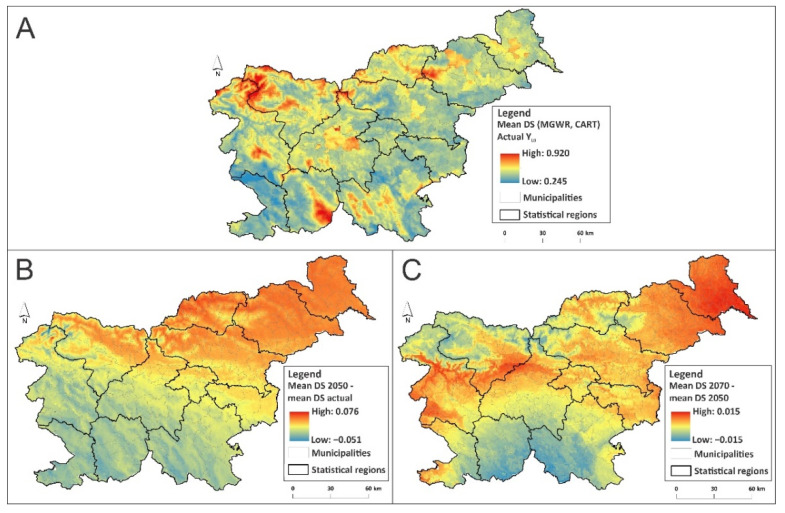
(**A**) Current LB infection risk map (MGWR and CART ensemble mean value); (**B**) infection risk difference under the RCP8.5 scenario between 2050 and the current state; (**C**) infection risk difference under the RCP8.5 scenario between 2070 and 2050. All future predictions are based on the mean values derived from five different global climate models (HadGEM2-ES, CCSM4, MIROC-ESM, HadGEM2-CC and MPI-ESM-LR).

Finally, we used both developed algorithms to downscale Y_LB_ values and prepare an LB infection risk map by considering currently realistic (RCP8.5) climate change predictions for the second half of the 21st century (2050 and 2070). Thus, the spatial resolution of the dependent variable was immensely improved, since we now operated with a grid of 30 arc seconds (~1 km^2^) instead of in individual municipalities. Figure 6A summarizes the current LB infection risk value resulting from the cumulative mean from the MGWR and CART models. However, future predictions indicate that the level of infection risk with LB will increase by 2050 in most parts of Slovenia (by up to 7.6%), especially in the northern and northeastern parts of the country (Figure 6B). Between 2050 and 2070, climate change could additionally increase LB infection risk (by up to 1.5%) in the central, western and eastern parts of Slovenia (Figure 6C). We can expect that by the end of the 21st century, the following regions will be severely affected: the western Mura Statistical Region, northern Gorizia and Upper Carniola Statistical Region, western Drava Statistical Region, and the northern Savinja and Carinthia Statistical Region.

## 4. Discussion

The fact that global warming can trigger shifts in species range distribution is already well known. Climate change affects tick habitat suitability and alters their spatial distribution [13,14,15,16]. It has a significant influence on tick hosts, which are crucial for their survival [11]. These processes leave behind distinct spatial patterns of tick-borne diseases [12]. Ostfeld and Brunner [11] reported that tick-borne diseases, such as LB and tick-borne meningoencephalitis, are already expanding over considerable areas of North America and Eurasia, since tick populations are moving to higher latitudes and altitudes. It seems that Slovenia is no exception; our results prove the same climate-change-triggered spatial trend in the case of LB. Therefore, our findings are even more alarming, since Slovenia is already among the leading countries for LB incidence [18]. It is predicted that these five LB foci could become larger due to climate change. LB infection risk will increase in the second half of the 21st century, particularly in the central, western and eastern parts of Slovenia. However, we learned from both models that other environmental factors (elevation, specific land use, host abundance) and socio-economic factors (population density, tourists, labor migration, education, etc.) are important co-creators of the LB footprint. Therefore, the developed infection risk map for the current and future climatic conditions in Slovenia has a major applicative value. Such products enable a more accurate assessment of the epidemiological situation, which leads to better preparation of mitigation plans and preventive action implementation [40]. Understanding the spatial patterns of human exposure to disease vectors, as well as related pathogens, is essential for limiting and controlling the prevalence of tick-borne diseases [41]. Moreover, LB already exerts an impact on employee health, travel choices, and the economic sustainability of tourism in endemic areas [42]. The same authors [42] concluded that theoretical and applicative research is needed to improve our knowledge of the relationships between public health, tourism, and the natural environment so that tourism management stakeholders can be empowered to be active agents in the evolving, transdisciplinary efforts to prevent, manage, and recover from Lyme disease outbreaks.

However, due to recent advances in geospatial information technology, infection risk maps can be easily integrated into web-based or mobile platforms to meet the needs of multiple customer groups (health care professionals, outdoor workers, resident populations and tourists) affected by and dealing with increasing levels of LB. The ESA demonstration project LymeApp (https://business.esa.int/projects/lymeapp (accessed on 9 October 2020)) is a good example of where geospatial modelling techniques were applied on Scottish Lyme data to produce maps of Lyme risk and vector distribution. Users were also able to report the locations of ticks and bites to the central database. If such spatial decision support and risk management systems covered all countries with a high LB prevalence/incidence, we could potentially limit the substantial productivity losses and decrease the economic and social costs caused by this disease on a global scale.

## 5. Conclusions

The climate crisis is deepening. Nowadays, the direct or indirect consequences of climate change are recognized in each corner of the world. Lifeforms are reacting (and adapting) to the changed environment, and so are the pathogens that they carry. The studies that investigate the spatial pattern dynamics of dangerous human diseases, should thus receive public attention. Indeed, such studies always provide applicative results, supporting strategic preventive action planning and decision making, either on local, regional (national), or even, global levels. From this perspective, the following conclusions can be drawn from our study: (1) In Slovenia, significant spatial foci of Lyme borreliosis are evident. (2) These can be explained and modelled with “proxy” predictor variables describing natural and anthropogenic processes in the target area. (3) Climate change will definitely potentiate the prevalence of Lyme disease in Slovenia and perhaps trigger a spatial shift of existing foci. Finally (4), it is expected that, infection risk could increase by up to 10% by the end of the century, especially in more elevated areas. However, these findings are calling for action since Lyme borreliosis is perhaps not a fatal disease [43], but can cause long-term symptoms that lead to limitations to daily life even after treatment [44], thus causing high societal cost [45].

## Supplementary Materials

The following are available online at https://www.mdpi.com/article/10.3390/ijerph182212061/s1, Table S1: Correlation matrix of independent variables, where green indicates a positive correlation above threshold value +0.6 and red indicates a negative correlation below threshold value −0.6, Table S2: Global Poisson regression results, test statistics of predictor variables and results of the Monte Carlo Test for Spatial Variability, Table S3: MGWR diagnostic information and summary statistics for MGWR parameter estimates, Figure S1: ART modeling text (A) and graphical output (B); PC2 = average value of the second main component of bioclimatic variables, PC1 = average value of the first main component of bioclimatic variables, LU 231 = land use (pastures), LU 5000 = land use (dry open land with a special vegetation cover), NDVI m = average value of the NDVI vegetation index.
