# Supplementary material for: The Lyme Borreliosis Spatial Footprint in the 21st Century: A Key Study of Slovenia
Source: Int J Environ Res Public Health. 2021 Nov 17;18(22):12061. doi: 10.3390/ijerph182212061 (PMC8619322; doi:10.3390/ijerph182212061)
Supplement: Supplementary file 1 [file ijerph-18-12061-s001.zip › ijerph-1379263-supplementary.pdf]

Table S1. Correlation matrix of independent variables, where green indicates a positive correlation above threshold value +0.6 and red indicates a negative correlation below threshold value -0.6.

	LU 311	LU 312	LU 313	LU 321	SWF	F1 RK	F2 RK	F1 P	F2 P	AI	MDI
DEM	0.26	-0.71	-0.55	-0.67	0.74	-0.44	-0.21	0.27	-0.10	-0.07	0.19
NDVI m	-0.16	-0.46	-0.49	-0.47	0.49	-0.27	-0.31	0.24	-0.18	-0.16	0.12
NDVI a	0.23	-0.02	0.21	0.00	0.06	0.13	0.00	-0.22	0.02	0.08	-0.02
PC1	0.03	-0.60	-0.37	-0.72	0.78	-0.55	0.04	0.31	-0.03	-0.05	0.32
PC2	0.46	-0.09	-0.17	0.18	-0.20	0.36	-0.50	-0.20	-0.09	0.09	-0.30
LU 1800	0.16	0.32	0.21	0.62	-0.51	0.45	0.04	-0.22	0.15	-0.16	-0.03
LU 1420	0.12	-0.31	0.05	-0.37	0.43	-0.34	0.14	0.09	0.04	0.25	0.07
LU 1300	0.16	-0.42	0.03	-0.40	0.68	-0.43	0.18	0.17	-0.05	0.19	-0.08
LU 1500	-0.07	-0.20	-0.10	-0.26	0.35	-0.36	0.11	0.14	-0.05	0.19	-0.02
LU 5000	0.14	0.24	0.06	0.53	-0.42	0.35	-0.16	-0.09	-0.10	-0.15	-0.23
LU 1222	0.16	-0.45	0.01	-0.50	0.71	-0.48	0.25	0.25	-0.11	0.11	-0.05
LU 122	0.02	-0.25	-0.29	-0.39	0.39	-0.28	-0.18	0.06	-0.26	0.19	-0.29
LU 231	-0.06	0.34	0.43	0.21	-0.18	0.03	0.40	-0.08	0.04	0.32	-0.31
LU 311	1.00	-0.54	-0.30	-0.06	0.16	0.09	-0.16	-0.05	0.10	-0.09	0.13
LU 312	-0.54	1.00	0.54	0.55	-0.72	0.28	0.25	-0.19	0.05	0.10	-0.19
LU 313	-0.30	0.54	1.00	0.27	-0.32	0.10	0.30	-0.20	0.13	0.25	-0.22
LU 321	-0.06	0.55	0.27	1.00	-0.71	0.48	0.15	-0.19	0.06	-0.17	-0.07
SWF	0.16	-0.72	-0.32	-0.71	1.00	-0.55	-0.05	0.28	-0.11	0.07	0.10
F1 RK	0.09	0.28	0.10	0.48	-0.55	1.00	-0.35	-0.53	-0.06	-0.07	-0.13
F2 RK	-0.16	0.25	0.30	0.15	-0.05	-0.35	1.00	0.17	0.22	-0.08	0.18
F1 P	-0.05	-0.19	-0.20	-0.19	0.28	-0.53	0.17	1.00	-0.42	-0.16	-0.03
F2 P	0.10	0.05	0.13	0.06	-0.11	-0.06	0.22	-0.42	1.00	0.04	0.56
AI	-0.09	0.10	0.25	-0.17	0.07	-0.07	-0.08	-0.16	0.04	1.00	-0.46
MDI	0.13	-0.19	-0.22	-0.07	0.10	-0.13	0.18	-0.03	0.56	-0.46	1.00

NDVI a
-0.03
-0.18
1.00
-0.03
0.21
0.05
0.25
0.20
-0.06
-0.05
0.16
-0.07
0.21
0.23
-0.02
0.21
0.00
0.06
0.13
0.00
-0.22
0.02
0.08
-0.02

	PC1	PC2	LU 1800	LU 1420	LU 1300	LU 1500	LU 5000	LU 1222	LU 122	LU 231
DEM	0.82	0.07	-0.61	0.21	0.33	0.19	-0.46	0.42	0.45	-0.48
NDVI m	0.52	-0.11	-0.45	-0.06	0.03	0.14	-0.18	0.04	0.36	-0.52
NDVI a	-0.03	0.21	0.05	0.25	0.20	-0.06	-0.05	0.16	-0.07	0.21
PC1	1.00	-0.42	-0.69	0.40	0.41	0.29	-0.64	0.50	0.28	-0.25
PC2	-0.42	1.00	0.23	-0.26	-0.21	-0.20	0.37	-0.27	0.15	-0.21
LU 1800	-0.69	0.23	1.00	-0.17	-0.12	-0.31	0.58	-0.24	-0.37	0.25
LU 1420	0.40	-0.26	-0.17	1.00	0.47	0.29	-0.42	0.49	-0.03	0.20
LU 1300	0.41	-0.21	-0.12	0.47	1.00	0.24	-0.19	0.88	0.10	0.28
LU 1500	0.29	-0.20	-0.31	0.29	0.24	1.00	-0.31	0.24	0.16	-0.01
LU 5000	-0.64	0.37	0.58	-0.42	-0.19	-0.31	1.00	-0.31	-0.06	0.01
LU 1222	0.50	-0.27	-0.24	0.49	0.88	0.24	-0.31	1.00	0.12	0.25
LU 122	0.28	0.15	-0.37	-0.03	0.10	0.16	-0.06	0.12	1.00	-0.24
LU 231	-0.25	-0.21	0.25	0.20	0.28	-0.01	0.01	0.25	-0.24	1.00
LU 311	0.03	0.46	0.16	0.12	0.16	-0.07	0.14	0.16	0.02	-0.06
LU 312	-0.60	-0.09	0.32	-0.31	-0.42	-0.20	0.24	-0.45	-0.25	0.34
LU 313	-0.37	-0.17	0.21	0.05	0.03	-0.10	0.06	0.01	-0.29	0.43
LU 321	-0.72	0.18	0.62	-0.37	-0.40	-0.26	0.53	-0.50	-0.39	0.21
SWF	0.78	-0.20	-0.51	0.43	0.68	0.35	-0.42	0.71	0.39	-0.18
F1 RK	-0.55	0.36	0.45	-0.34	-0.43	-0.36	0.35	-0.48	-0.28	0.03
F2 RK	0.04	-0.50	0.04	0.14	0.18	0.11	-0.16	0.25	-0.18	0.40
F1 P	0.31	-0.20	-0.22	0.09	0.17	0.14	-0.09	0.25	0.06	-0.08
F2 P	-0.03	-0.09	0.15	0.04	-0.05	-0.05	-0.10	-0.11	-0.26	0.04
AI	-0.05	0.09	-0.16	0.25	0.19	0.19	-0.15	0.11	0.19	0.32
MDI	0.32	-0.30	-0.03	0.07	-0.08	-0.02	-0.23	-0.05	-0.29	-0.31

	DEM	NDVI m
DEM	1	0.55
NDVI m	0.55	1.00
NDVI a	-0.03	-0.18
PC1	0.82	0.52
PC2	0.07	-0.11
LU 1800	-0.61	-0.45
LU 1420	0.21	-0.06
LU 1300	0.33	0.03
LU 1500	0.19	0.14
LU 5000	-0.46	-0.18
LU 1222	0.42	0.04
LU 122	0.45	0.36
LU 231	-0.48	-0.52
LU 311	0.26	-0.16
LU 312	-0.71	-0.46
LU 313	-0.55	-0.49
LU 321	-0.67	-0.47
SWF	0.74	0.49
F1 RK	-0.44	-0.27
F2 RK	-0.21	-0.31
F1 P	0.27	0.24
F2 P	-0.10	-0.18
AI	-0.07	-0.16
MDI	0.19	0.12

DEM=digital elevation model (25 m horizontal resolution) (e-Geodetic data online platform) NDVI m=average value of the NDVI vegetation index (EarthData database); NDVI a=linear trend coefficient of the NDVI vegetation index (EarthData database); PC1=average value of the first main component of bioclimatic variables (CHELSA database); PC2=average value of the second main component of bioclimatic variables (CHELSA database); LU 1800=land use (agricultural land overgrown with forest trees) (MKGP-portal database); LU 1420=land use (forest tree plantation) (MKGP-portal database); LU 1300=land use (permanent meadow) (MKGP-portal database); LU 1500=land use (trees and shrubs) (MKGP-portal database); LU 5000=land use (dry open land with a special vegetation cover) (MKGP-portal database); LU 1222=land use (extensive or meadow orchard) (MKGP-portal database); LU 122=land use (urban areas with green patches) (Land Copernicus database); LU 231=land use (pastures) (Land Copernicus database); LU 311=land use (deciduous forest) (Land Copernicus database); LU 312=land use (coniferous forest) (Land Copernicus database); LU 313=land use (mixed forest) (Land Copernicus database); LU 321=land use (natural grasslands) (Land Copernicus database); SWF=land use (small woody features) (Land Copernicus database); F1 RK=first factor of roadkill (Slovenia Forest Service database); F2 RK=second factor of roadkill (Slovenia Forest Service database); F1 P=first demographic factor (STAGE database, GOV.SI database and SiStat database); F2 P=second demographic factor (STAGE database, GOV.SI database and SiStat database); AI=population aging index (STAGE database); MDI=municipal development index (GOV.SI database).

Table S2. Global Poisson regression results, test statistics of predictor variables and results of the Monte Carlo Test for Spatial Variability.

Deviance	1143.30
Log-likelihood	-1107.58
AIC	2249.16
AICc	1180.45
Percent deviance explained	0.21
Adj. percent deviance explained	0.14

Variable	Est.	SE	t(Est/SE)	p-value	Spatial Variability p-value
Intercept	2.01	0.34	5.97	0.000	0.002
DEM	-1.10	0.31	-3.54	0.000	0.000
PC1	2.05	0.29	7.07	0.000	0.002
PC2	1.09	0.31	3.50	0.000	0.000
LU 1800	0.58	0.11	5.27	0.000	0.015
LU 1300	-0.53	0.11	-4.78	0.000	0.069
LU 1500	0.79	0.20	4.05	0.000	0.232
LU 5000	0.26	0.08	3.17	0.002	0.008
LU 122	-0.29	0.07	-3.97	0.000	0.567
LU 231	0.39	0.11	3.68	0.000	0.648
LU 311	-0.47	0.09	-5.06	0.000	0.066
LU 313	-0.22	0.11	-2.01	0.044	0.000
LU 321	-0.44	0.08	-5.39	0.000	0.007
SWF	0.95	0.15	6.30	0.000	0.007
F1 RK	0.04	0.02	2.82	0.005	0.906
F2 P	-0.08	0.01	-5.35	0.000	0.266
MDI	-0.79	0.17	-4.75	0.000	0.001

DEM=digital elevation model (25 m horizontal resolution); PC1=average value of the first main component of bioclimatic variables; PC2=average value of the second main component of bioclimatic variables; LU 1800=land use (agricultural land overgrown with forest trees); LU 1300=land use (permanent meadow); LU 1500=land use (trees and shrubs); LU 5000=land use (dry open land with a special vegetation cover); LU 122=land use (urban areas with green patches); LU 231=land use (pastures); LU 311=land use (deciduous forest); LU 313=land use (mixed forest); LU 321=land use (natural grasslands); SWF=land use (small woody features); F1 RK=first factor of roadkill; F2 P=second demographic factor; MDI=municipal development index.

Table S3. MGWR diagnostic information and summary statistics for MGWR parameter estimates.

Effective number of parameters (trace(S))	78.95
Degree of freedom (n-trace(S))	133.05
Deviance	505.10
AIC	663.00
AICc	758.61
BIC	928.01
Adj. alfa (95 %)	0.01
Adj. critical t value (95 %)	2.57

Variable	Mean	STD	Min	Median	Max
Intercept	2.28	3.72	-8.84	2.93	10.81
DEM	-2.51	5.41	-19.17	-2.37	6.02
PC1	1.78	3.71	-4.96	2.30	11.29
PC2	1.97	4.99	-7.04	2.18	17.15
LU 1800	-0.11	0.80	-1.97	0.15	0.81
LU 1300	-0.97	0.76	-2.22	-0.95	1.15
LU 1500	0.64	1.13	-1.42	0.76	2.57
LU 5000	0.24	0.75	-1.47	0.27	1.74
LU 122	-0.53	0.37	-1.85	-0.46	0.28
LU 231	0.47	0.48	-0.18	0.29	2.08
LU 311	-0.41	0.63	-1.97	-0.38	1.09
LU 313	0.19	2.22	-4.01	-0.60	5.40
LU 321	-0.83	0.72	-2.04	-0.92	0.75
SWF	1.43	2.84	-10.79	1.43	9.69
F1 RK	0.03	0.05	-0.06	0.03	0.16
F2 P	-0.05	0.07	-0.20	-0.06	0.09
MDI	-0.62	1.45	-3.86	-0.78	1.79

DEM=digital elevation model (25 m horizontal resolution); PC1=average value of the first main component of bioclimatic variables; PC2=average value of the second main component of bioclimatic variables; LU 1800=land use (agricultural land overgrown with forest trees); LU 1300=land use (permanent meadow); LU 1500=land use (trees and shrubs); LU 5000=land use (dry open land with a special vegetation cover); LU 122=land use (urban areas with green patches); LU 231=land use (pastures); LU 311=land use (deciduous forest); LU 313=land use (mixed forest); LU 321=land use (natural grasslands); SWF=land use (small woody features); F1 RK=first factor of roadkill; F2 P=second demographic factor; MDI=municipal development index.

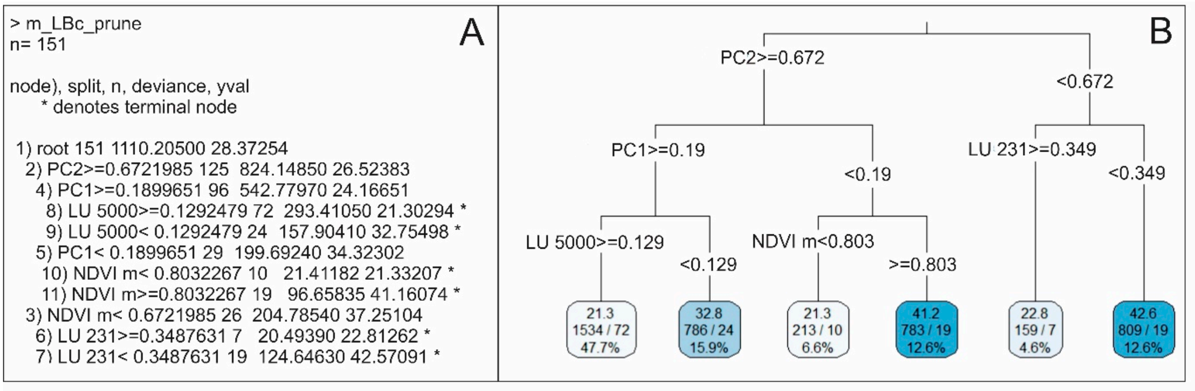

Figure S1. (**A**) CART modeling text; (**B**) CART graphical output; PC2=average value of the second main component of bioclimatic variables, PC1=average value of the first main component of bioclimatic variables, LU 231=land use (pastures), LU 5000=land use (dry open land with a special vegetation cover), NDVI m=average value of the NDVI vegetation index.
